# Metavirome datasets from two endemic Baikal sponges *Baikalospongia bacillifera*

**DOI:** 10.1016/j.dib.2020.105260

**Published:** 2020-02-07

**Authors:** Tatyana V. Butina, Igor V. Khanaev, Lyubov S. Kravtsova, Olga O. Maikova, Yurij S. Bukin

**Affiliations:** a Limnological Institute, Siberian Branch of Russian Academy of Sciences, 3, Ulan-Batorskaya Street, Irkutsk, 664033, Russia; b Irkutsk State University, Faculty of Biology and Soil Studies, 5, Sukhe-Bator Street, Irkutsk, 664011, Russia

**Keywords:** Metagenomics, Viral communities, Viral diversity, Virome, Sponge holobiont, Freshwater sponges, Lake Baikal

## Abstract

Sponges are ecologically important components of marine and freshwater benthic environments; these holobionts contain a variety of microorganisms and viruses. For the metagenomic characterization of potential taxonomic and functional diversity of sponge-associated dsDNA viruses, we surveyed two samples of Baikal endemic sponge *Baikalospongia bacillifera* (diseased and visually healthy). In total, after quality processing, we have obtained 3 375 063 and 4 063 311 reads; of these 97 557 and 88 517 sequences, accounting for ca. 2.9 and 2.2% of datasets, have been identified as viral. We have revealed approximately 28 viral families, among which the bacteriophages of the *Myoviridae*, *Siphoviridae* and *Podoviridae* families, as well as the viruses of the *Phycodnaviridae* and *Poxviridae* families, dominated in the samples. Analysis of viral sequences using the COG database has indicated 22 functional categories of proteins. Viral communities of visually healthy and diseased Baikal sponges were significantly different. The metagenome sequence data were deposited to NCBI SRA as BioProject PRJNA577390.

**Table:** Specifications Table

Subject	Biology
Specific subject area	Metagenomics
Type of data	Table Figures Metagenome sequences of viruses
How data were acquired	Shotgun DNA sequencing using Illumina MiSeq
Data format	Raw data, analyzed
Parameters for data collection	Two individuals (diseased and visually healthy) of endemic Baikal sponge *Baikalospongia bacillifera*
Description of data collection	The *Baikalospongia bacillifera* sponges of 5–7 cm^3^ in volume were sampled from Lake Baikal using lightweight diving equipment in May 2018 at depths of 16–20 m. One sample looked healthy, and another had necrosis lesions
Data source location	Country: Russia Region: Lake Baikal Latitude and longitude for collected samples: 51°54′07.5″N, 105°06′12.0″E
Data accessibility	Raw data were deposited to NCBI Repository name: SRA Data identification number: BioProject PRJNA577390, BioSamples SAMN13025046 and SAMN13025227 Direct URL to data: https://www.ncbi.nlm.nih.gov/sra/PRJNA577390

**Table:** 

**Value of the Data** • These are the first metavirome data on the freshwater sponges *Baikalospongia bacillifera.* • The data provides valuable information about the diversity and functional potential of dsDNA viral communities in the sponge holobionts. • This data is useful for comparing viral communities in different marine and freshwater sponges. • Raw sequence data can be used for various additional bioinformatics processing. • The data can be used for investigations of sponge diseases

## 1 Data

Sponges are a highly complex system that comprises a variety of microorganisms and viruses [1]. The diversity and the roles of sponge-associated viruses have been little known compared to those of other members of the sponge holobiont [[2], [3], [4], [5]].

Here, we present two virome datasets (dsDNA viral sequences) from freshwater sponges *Baikalospongia bacillifera.* One sponge had necrosis lesion (Sv2475) and other was visually healthy (Sv2478). The raw data contained 3 842 088 and 5 035 528 pair sequence reads for the samples Sv2475 and Sv2478, respectively. After quality processing of data, we have obtained 3 375 063 and 4 063 311 reads, ranging from 80 to 256 bp. Of them, 97 557 and 88 517 sequences were identified as viral using the NCBI RefSeq viral genomes database (e-value ≤ 10^−5^; bit score ≥ 50), accounting for ca. 2.9 and 2.2% of datasets.

**Fig. 1:**
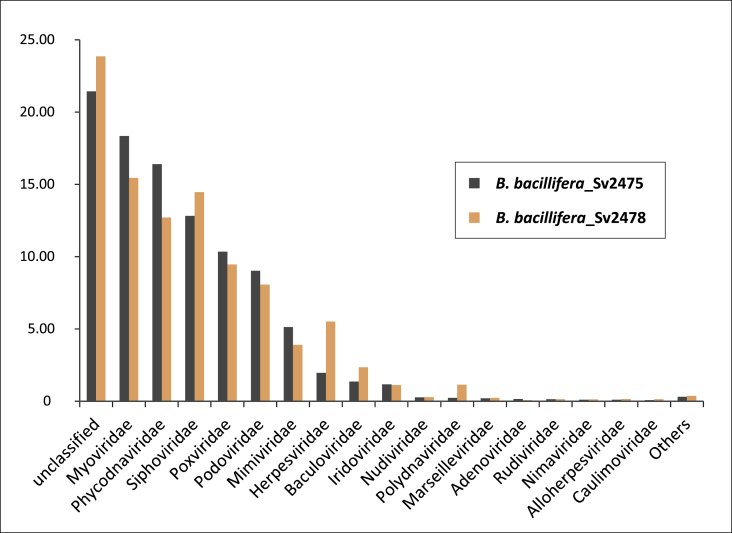
The proportion of identified DNA viral families and viruses that were unclassified at the family rank.

The families *Myoviridae*, *Phycodnaviridae*, *Siphoviridae*, *Poxviridae*, *Podoviridae*, *Mimiviridae*, *Herpesviridae*, *Baculoviridae,* and *Iridoviridae* were the most numerous, represented more than 1% of the sequences and in total accounted for more than 70% of the identified virome sequences. (Fig. 1). We did not classify the significant parts of viral reads (21.4% and 23.9% in the samples Sv2475 and Sv2478, respectively) at the family rank.

The diversity, richness and difference of two viral communities were estimated using Shannon, Simpson, ACE and Chao1 indices (Table 1), rarefaction technique and chi-square test. The rarefaction curves for the both samples reached a plateau (data not shown). Viral communities of visually healthy and diseased Baikal sponges were significantly different (p-value < 2.2e-16).

**Table 1:** Biodiversity and richness indices for the virome datasets.

Samples	Shannon index	Simpson index	Alpha diversity	Chao1	ACE
**Sv 2475**	5.26145	0.98274	986	986	986
**Sv 2478**	5.35669	0.98464	973	973	973

**Fig. 2:**
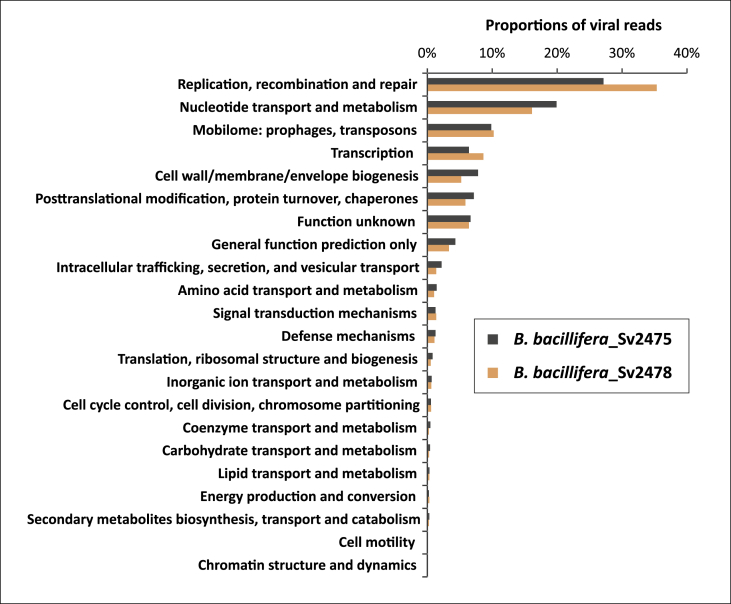
The percentage of viral sequences associated with the general COG functional categories.

The comparison of revealed viral reads with the COG database has indicated the 22 functional categories of proteins and enzymes (Fig. 2). Of them, the most representative (more than 5%) were proteins of replication, recombination and repair, nucleotide transport and metabolism, and mobile genomic elements (prophages and transposons).

This is the first report on the diversity of dsDNA viral communities in endemic Baikal sponges *B. bacillifera* based on Illumina MiSeq sequencing approach. Datasets were deposited to SRA NCBI database: SRA accession PRJNA577390.

## 2 Experimental design, materials, and methods

### 2.1 Sampling and isolation of viral DNA

The endemic Baikal sponges *Baikalospongia bacillifera* were sampled in the southern basin of Lake Baikal (near Bolshiye Koty, 51°54′07.5″N, 105°06′12.0″E) at depths of 16–20 m in May 2018 using lightweight diving equipment. Two individuals of *B. bacillifera* of 5–7 cm^3^ in volume were collected: one looked healthy (Sv2478), and another had necrosis lesions (Sv2475). The sponge samples were twice washed in sterile Baikal water and thoroughly homogenized using a blender. Then homogenates were frozen in nitrogen and transported to the laboratory. The samples were gently thawed, twice diluted with SM buffer (0.2 M NaCl; 10 mM MgSO_4_; 50 mM Tris HCl, pH 7.5), shaken with a Heidolph Multi Reax Vortex Mixer (10,000 rpm, 30 min), and were centrifuged 400 g for 15 min followed by 16,000 g for 30 min. The aqueous fraction was passed through a syringe filter with a pore size of 0.2 μm (Sartorius) and treated with DNase I (50 U/ml) and RNase A (100 μg/ml) enzymes (Thermo Fisher Scientific) to remove contaminating nucleic acids. Viral DNA was extracted by ZR Viral DNA kit (Zymo Research).

### 2.2 Library preparation and sequencing

The preparation and sequencing of DNA libraries were performed in The Center of Shared Scientific Equipment “Persistence of microorganisms” of Institute for Cellular and Intracellular Symbiosis UB RAS, Russia. The paired-end libraries were prepared using a NEBNext Ultra II FS DNA Library Prep Kit for Illumina (NEB) according to the manufacturer's protocol. The validation of DNA libraries was verified by Agilent 2100 Bioanalyzer (Agilent Technologies). Sequencing of the libraries was conducted on a MiSeq genome sequencer using MiSeq Reagent Kit v3 (2х300cycles, Illumina).

### 2.3 Analysis of virome datasets

The primary processing (quality control and trimming) of the metavirome datasets (paired reads of 2 × 300 bp) was performed using the R package “ShortReads” [6]. The first (up to 15) and last (up to 30) nucleotides with low quality were removed. The sequences of less than 80 nucleotides were excluded from datasets.

Taxonomic identification of viral sequences was performed using the BLASTn algorithm [7] against NCBI RefSeq viral complete genomes database (September 2018 release) [8]. The BLASTn parameters used were as follows: cost to open a gap, two; cost to extend a gap, one; word size for word finder algorithm, twelve; penalty for a nucleotide mismatch, one; the reward for a nucleotide match, one. The sequence reads were considered ‘identified’ if they had a relative in the reference database with an e-value of ≤10^−5^ and bit score ≥50. The BLASTn analysis data were saved as a hit table. BLAST hits corresponding to the same viral genome subject ID were considered to belong to one virotype. Each subject ID from the BLASTn hit table was converted to a taxonomic annotation.

For the functional annotation of viral sequences, we used the local Blastx application [7] and COG database [9]. The BLASTx parameters used were as follows: cost to open a gap, six; cost to extend a gap, two; word size for word finder algorithm, six; e-value of ≤10^−5^ and bit score ≥50.

Rarefaction analysis was performed to assess the species richness in the samples [10]. The Shannon, Simpson, ACE and Chao1 indices were calculated for assessing the diversity of viral communities [11]. The reliability of the difference between two *B. bacillifera* viral communities was estimated using the chi-square test. Statistical calculations were performed using the R packages “vegan” [12] and “pvclust” [13].
